# Cameroon mid-level providers offer a promising public health dentistry model

**DOI:** 10.1186/1478-4491-10-46

**Published:** 2012-11-26

**Authors:** Leo Ndiangang Achembong, Agbor Michael Ashu, Amy Hagopian, Ann Downer, Scott Barnhart

**Affiliations:** 1 Department of Global Health, University of Washington, Seattle, WA 98105, USA; 2 Cameroon Baptist Convention Health Board, P.O. Box 1, Nkwen-Bamenda, NWP, Cameroon

**Keywords:** Mid-level dental providers, Oral health, Dental care, Health workforce, Task shifting, Rural health care, Africa, Cameroon

## Abstract

**Background:**

Oral health services are inadequate and unevenly distributed in many developing countries, particularly those in sub-Saharan Africa. Rural areas in these countries and poorer sections of the population in urban areas often do not have access to oral health services mainly because of a significant shortage of dentists and the high costs of care. We reviewed Cameroon’s experience with deploying a mid-level cadre of oral health professionals and the feasibility of establishing a more formal and predictable role for these health workers. We anticipate that a task-shifting approach in the provision of dental care will significantly improve the uneven distribution of oral health services particularly in the rural areas of Cameroon, which is currently served by only 3% of the total number of dentists.

**Methods:**

The setting of this study was the Cameroon Baptist Convention Health Board (BCHB), which has four dentists and 42 mid-level providers. De-identified data were collected manually from the registries of 10 Baptist Convention clinics located in six of Cameroon’s 10 regions and then entered into an Excel format before importing into STATA. A retrospective abstraction of all entries for patient visits starting October 2010, and going back in time until 1500 visits were extracted from each clinic.

**Results:**

This study showed that mid-level providers in BCHB clinics are offering a full scope of dental work across the 10 clinics, with the exception of treatment for major facial injuries. Mid-level providers alone performed 93.5% of all extractions, 87.5% of all fillings, 96.5% of all root canals, 97.5% of all cleanings, and 98.1% of all dentures. The dentists also typically played a teaching role in training the mid-level providers.

**Conclusions:**

The Ministry of Health in Cameroon has an opportunity to learn from the BCHB model to expand access to oral health care across the country. This study shows the benefits of using a simple, workable, low-cost way to provide needed dental services across Cameroon, particularly in rural areas.

## Introduction

Cameroon, like other sub-Saharan African countries, experiences a significant shortage of licensed, trained health personnel [1]. Oral health services are inadequate in many developing countries, particularly those in sub-Saharan Africa [2,3]. The few available dentists in sub-Saharan African countries are located in urban areas, leaving most people with very limited access to affordable oral health care [4]. While most of the international focus on health workforce problems is aimed at the shortage of physicians, nurses, and midwives, there is also a significant deficit of dental health professionals. Even though there have been significant improvements in oral health in many countries, it is anticipated that developing countries will experience an increased incidence of dental caries secondary to the broadened availability of sugar-loaded beverages, other dietary changes, and inadequate attention to preventive measures [5,6]. Some determinants of general health, including diet, hygiene, smoking, alcohol use, stress, and trauma, are associated with obesity, diabetes, cancers, and cardiovascular diseases, and are also associated with oral health [7]. Oral manifestations of HIV are also having a negative effect on the quality of life [8].

The use of mid-level dental providers (MLPs), who require less education and whose salaries are lower than fully trained dentists, offers a task-shifting approach to the dental workforce crisis. In the Netherlands, dental hygienists are moving beyond cleanings and oral health education to providing sealants, fillings, and simple extractions for children [9]. In the USA, both Alaska and Minnesota have licensed MLPs to perform some basic dentistry [10]. Other states are also looking at recommendations to deploy a mid-level dental workforce, especially to address the needs of children and rural communities [11]. Only 11 sub-Saharan African countries had dental schools training dentists in 2000, and only six countries had any sort of training for dental auxiliaries The purpose of this paper was to explore the dental care provided by existing dental MLPs in Cameroon, all of them employed by the Cameroon Baptist Convention Health Board (BCHB), while evaluating their efficacy and productivity, to inform the policy decision of whether to expand dental MLP services in the nation.

To explore dental practice patterns within BCHB dental clinics, we conducted a descriptive study to: identify the numbers and types of procedures done by dentists and MLPs; compare the practice patterns of dentists and MLPs; compare practice patterns of clinics with MLPs and dentists and clinics with only MLPs; and estimate the number of MLPs and dentists needed to provide reasonable access to dental care.

## Methods

### Setting

#### Cameroon Baptist Convention Health Board

The largest oral health provider in Cameroon today is the private, non-profit BCHB. The BCHB operates 10 dental clinics located in six of Cameroon’s 10 regions. Four of these clinics have resident dentists and the other six are run mostly by MLPs. New MLPs are trained in the BCHB private training school for health personnel when there is a need for MLPs within the organization. All MLPs are trained by and to work only in BCHB clinics. Dental MLPs in this study included seven dental therapists and 35 dental assistants. The therapists (most of whom were former dental assistants) completed a 3-year course and the assistants completed a 1-year course. Both groups are high school graduates in science subjects. Dental therapist training included 2 years of classroom work with lectures on basic medical sciences related to dentistry (anatomy, physiology, and biochemistry); basic oral surgery (extractions and management of minor facial injuries); periodontics; restorative dentistry; endodontics; and prosthetics (partial and complete removable dentures). The third year is spent in the clinics.

The dental therapists were functioning at a very high skill level, performing complex dental procedures like root canals, and managing facial injury. The dental assistants, who complete a fast-track 1-year course focused on the BCHB’s community oral health programme, generally start off performing simple fillings and extractions, but gradually acquire skills to perform some of the more difficult procedures. The MLPs are given sufficient opportunities to hone their skills under direction of the dentists. Even though the Cameroon government recognizes this school, there is presently no licensure for MLPs by the Cameroon National Dental Council. The four clinics with dentists are also supported by BCHB-trained dental MLPs, who provide a significant amount of services. The four fully trained Cameroonian dentists staffing the clinics were all licensed to practice dentistry. Two of them were trained in Nigeria, the other two in Russia.

### Participant observation

An author of this paper (LNA) personally observed activities and the functioning of these clinics while working for the BCHB from March 2004 to September 2009 as the dentist in one of the clinics and also as supervisor of dental services for all 10 dental clinics.

### Data collection

Following human subjects research approval at the University of Washington and the BCHB, secondary data were collected from the registries of 10 dental clinics by 10 dental assistants, under the supervision of four dentists, who were collaborators on this study. Only the dental assistants and their supervising dentists had access to confidential patient information.

In Cameroon, paper registries are used for all patient services. These registries were ledgers with a column for visit number, patient name, address, diagnosis, separate columns for different procedures (a check was placed in each procedure column for that patient visit), and provider column where the provider would write their name and a final column for cost. Each date was noted in the ledger. For this paper, the following information was abstracted: visit number; procedure(s) performed (e.g. extraction, filling, sealants, minor facial injury major facial injury, root canal treatment, dentures, and cleaning); the provider type who performed the procedure (determined by the name of the provider); and date the procedure was performed.

The sample used was a retrospective abstraction of all entries for patient visits starting in October 2010, and going back in time until 1500 visits were extracted from each clinic (some data extractors reported a few more than 1500 visits; these extra data points were not removed). Some clinics were busier than others, so the 1500-patient visit count was reached in a shorter time in some locations. Clinic 5 only began treating dental patients in December 2008, and so had only 970 patient visits in the study, short of the 1500 entries sought. Data were collected manually on a tally sheet and then entered into an Excel format before importing into STATA [12]. The number of entries as 1500 in each clinic was based on the unit of visits, not on individual patients. The registries used were thus to identify a continuous retrospective sample of 1500 patient care visits, but fewer than 1500 patients were captured because of repeat visits. Six of the clinics were staffed only by MLPs, while four other clinics had both MLPs and one dentist each. The 10 dental clinics were de-identified and assigned numbers (1 through 10).

## Results

There were 42 MLPs and four dentists serving across the 10 dental clinics. Clinics 4, 5, 6, 7, 8, and 9 employed only MLPs. All clinics reported complete data for extractions, fillings, minor facial injuries, major facial injuries, cleanings, and dentures. There was a failure to capture data for root canals and dentures in Clinic 1. Sealants were done only in Clinic 1. Major facial injuries were treated only in Clinics 1, 3, and 7. Dentures were not offered in Clinics 4 and 8. Clinic 5 is the newest one, with only 90 patient visits at the point of our data collection. Extractions are by far the most common procedures in these clinics.

Table 1 illustrates the autonomy of MLPs, who alone performed 94% of all extractions, 88% of all fillings, 97% of all root canals, 98% of all cleanings, and 98% of all dentures. Major facial injuries, and, to a lesser extent, minor facial injuries, are mostly managed by the dentists.

**Table 1:** **Procedures performed by dentists, MLPs, or both in Baptist convention clinics, Cameroon, Africa**^**a**^

	**Dentist**	**MLP**	**Both**	**Total**
Extractions	154 (4.2%)	3433 (93.8%)	71 (1.9%)	3658 (99.9)
Fillings	233 (10.7%)	1906 (88.2%)	20 (0.9%)	2159 (99.8)
Root canals	63 (3.4%)	1762 (96.5%)	0 (0%)	1825 (99.9)
Minor facial injuries	84 (35.1%)	152 (63.5%)	3 (1.2%)	239 (99.8)
Major facial injuries	30 (78.9%)	6 (15.7%)	2 (5.2%)	38 (99.8)
Cleanings	10 (1.4%)	679 (97.5%)	7 (1%)	696 (99.9)
Dentures	7 (1.8%)	375 (98.1%)	0 (0%)	382 (99.9)
Sealants	8 (10.2%)	68 (87.1%)	2 (2.5%)	78 (99.8)
Total	589 (6.4)	8381 (92.3)	105 (1.1)	9075 (99.8)

MLP, mid-level dental provider. ^a^Data presented as number (percentage).

*Source of data for Table*1: Analysis of 1500 patient visits to each of 10 Cameroonian dental clinics operated by the Baptist Convention, October 2010.

Table 2 illustrates the number of monthly patient visits for each clinic in 2010. The volume of annual visits ranged from 443 to 11 015 per clinic.

**Table 2:** Patient visits, showing a total number of 38 844 visits in 2010

**Clinic number**	**Number of MLPs on staff**	**Number of dentists on staff**	**Jan.**	**Feb.**	**Mar.**	**Apr.**	**May**	**Jun.**	**Jul.**	**Aug.**	**Sept.**	**Oct.**	**Nov.**	**Dec.**	**Total / average**
1	9	1	586	763	539	632	465	507	598	465	667	628	612	563	7 025/585.4
2	9	1	758	1129	2562	974	714	770	878	80	938	829	728	655	11 015/917.9
3	5	1	423	373	335	442	384	373	338	463	423	374	364	349	4 641/386.75
4	2	0	62	82	67	105	121	112	118	99	56	130	95	112	1 159/96.5
5	2	0	23	59	46	49	45	25	23	41	12	38	36	46	443/531.6
6	2	0	145	150	118	185	136	149	152	145	176	165	212	130	1 863/155.25
7	5	0	279	290	303	289	335	296	351	388	290	344	337	370	3 872/322.6
8	2	0	84	77	64	45	70	95	77	105	75	59	105	95	951/79.2
9	3	0	105	102	133	151	134	123	132	165	149	129	147	146	1 616/134.6
10	3	1	472	489	416	525	517	562	543	619	576	521	479	540	6 259/521.5
Total	42	4	2937	3514	4583	3397	2921	3012	3210	2570	3362	3217	3115	3006	38 844
Average	4.2	0.4	293.7	351.4	458.3	339.7	292.1	301.2	321	257	336.2	321.7	311.5	300.6	3 884.4

*Source of data for Table*2: Reports from 10 Cameroonian dental clinics operated by the Baptist Convention on their annual patient visit volumes.

### Urban/rural differences

Clinics 1, 2, 3, and 10, all in urban locations, have both MLPs and dentists. No rural clinics have dentists. Compared with the clinics without dentists, the urban clinics with dentists examined and treated more patients. We are unable to judge whether higher clinic volumes were attributable to the presence of a dentist or due to a higher population density.

### Leaving without treatment

While 14 514 patient visits were recorded in our data base for the 10 clinics, almost one-half (6335) left without any treatment procedures. Clinics 4 and 8 have the highest number of untreated patients, while Clinics 1 and 5 had the fewest untreated patients.

### Fillings, extractions, and root canals

There is a general trend of relatively few fillings done across the clinics except for Clinic 6, where 45% of visits are for fillings. This clinic is located in an urban area, and it has a higher number of patients having restorative procedures done with a lower percentage of extractions (19.3%). Extractions are done fairly frequently in BCHB dental clinics. Extractions were the most frequently performed procedures across the clinics, averaging 367 extractions (25.7%).

Clinics 2 and 6 show a consistent trend of a higher proportion of patients having procedures, largely restorative. The patients coming to these two clinics seem to be avoiding extractions in favour of more tooth restoration. Almost one-half the procedures in Clinic 2 are for root canals.

### Minor and major facial injuries

Clinics 1, 2, and 3 (those with dentists) manage most of the minor facial injuries in our sample, although all clinics (except for Clinic 8) manage some of these. Only Clinics 1, 2, and 7, who also have dentists present, recorded management of major facial injuries. Most of the major facial injuries presenting to these clinics are various types of mandibular fractures, typically involving reduction and immobilization of the jaws.

### Preventive care

Cleanings and sealants are important dental preventive measures. Unfortunately, of the patients treated in all BCHB dental clinics, few had cleanings. Of 14 514 visits, only 5% (696) were for tooth cleaning. Clinic 1 was the only clinic doing sealants, although only 78 patients in our sample (5.2%) received them.

## Discussion

Dental MLPs offer the vast majority of dental care in Cameroon’s BCHB dental services clinics. The data suggest that MLPs are offering a full scope of dental work across the 10 clinics, with the exception of treatment for major facial injuries. When clinics have at least one dentist along with mid-level providers, however, their productivity climbs considerably. The dentists in these clinics have typically also played a teaching role in training the MLPs, especially those newly graduated from training.

### Urban versus rural clinic differences

The proportionately higher number of fillings in urban clinics gives some indication that urban dweller accessibility to dental clinics is potentially associated with better oral health, better dental hygiene knowledge, and more financial resources. While rural clinics serve largely poor populations, urban clinics serve a slightly better-off population. Urban patients who are closer to the clinics are also more likely to come back for appointments. The cost and difficulty of travelling long distances to clinics is enormous.

### No treatments

We found one-half of potential dental patients leaving their visits with no recorded treatment or procedure. Oral medicine cases that usually require medications such as antibiotics, mouthwashes, or other modes of treatment are recorded as no procedure. Some of the reasons for the untreated patients may be: provision of non-procedural care such as antibiotics; patients coming for check-ups with no significant findings warranting treatments; lack of money to pay for treatment; minimal flexibility in pricing by providers (lower fees for poorer patients could encourage some patients to have treatment); patients postponing treatments because pain has not yet set in; and clinics with only MLPs referring patients to dentists in other clinics with complex problems.

### Scope of services

Extractions are by far the most common procedures in these clinics. The reasons for this may include: late presentation of patients with grossly carious teeth; many patients cannot afford the cost of a root canal; and ignorance by some patients regarding root canals. These patients insist on extractions only.

Patients coming to Clinics 2 and 6, where more root canals were done, seem to be avoiding extractions in favour of more tooth restoration. Almost one-half of the procedures in Clinic 2 are for root canals, although they are expensive and generally out of the reach of the average Cameroonian. Performing root canals without crowns tends to result in fracture over time [13]. Further study of the prognosis of root-filled teeth in these clinics is advisable.

Dentists typically are treating minor facial injuries around the oro-facial region. Dental workers in the BCHB and most of Cameroon are generally expected to be able to handle bruises, injuries to the lips and elsewhere around the face and inside the mouth, as well as dento-alveolar fractures. In a country where there is not a single certified maxillo-facial surgeon, and where other surgeons involved with managing trauma are scarce, the services provided by the BCHB are filling a gap. Clinic 1, with 31 major facial injuries, has become a referral centre for these kinds of injuries with patients coming from many different parts of Cameroon. Motorcycle accidents are one of the leading causes of these common injuries.

### Preventive care

The lack of preventive oral health care, such as tooth cleaning and sealant procedures, may be attributable to the cost to patients, and the low priority for cleaning among providers and patients alike. Only 78 sealants were applied among the visit sample. While acknowledged as an important preventive care measure in the dental literature [14], sealants are apparently not a priority in these 10 clinics. This is a cause for concern in a country where prevention should be the cornerstone for addressing oral health. Dental workers should be focusing on continuous oral health education with emphasis on prevention.

### Paying for care

There is no universal health insurance scheme in Cameroon, although in public facilities the cost is minimal. Only about 1% of the population, those who work in large privately owned companies, have any kind of health insurance. The BCHB dental clinics charge about 25,000CFA(approximately $50) for root canals. The minimum wage in Cameroon is 28 000 CFA (approximately $56) per month. Private dental clinics in the two biggest urban areas of Cameroon (Yaounde and Douala) offer root canals for 75 000 CFA (approximately $150), completely out of the reach for even wealthy Cameroonians.

### Oral health workforce

In 1999, sub-Saharan Africa had only 10 078 dentists, 2576 dental auxiliaries, and 682 other oral health personnel [15]. Given the sub-Saharan Africa population of 1.1 billion [16], each dentist would need to care for a population of nearly 100 000 people (99 226). By comparison, the USA had 186 084 dentists in 2009, for a ratio of 1:1642 [17]. African needs for oral health and emergency dental pain relief are clearly not being met.

The World Health Organization (WHO) advocates that oral health services be made more accessible in low-income countries using a primary health-care approach, with MLPs providing basic dental services [2]. In many countries where MLPs have been used to provide dental care, there is indication of progress toward accessibility of oral care, along with decreasing prevalence of dental caries [18].

The Basic Package of Oral Care, recommended by the WHO especially for low-income countries and disadvantaged populations, stresses the importance of using allied dental personnel in providing emergency oral services, oral health education, and atraumatic restorative treatment [19]. The WHO’s basic package consists of oral urgent treatment, affordable fluoride toothpaste, and atraumatic restorative treatment. This package enables the provision of dentistry using a primary care approach [2,19].

Cameroon has 172 registered practicing dentists, putting the dentist-to-population ratio at one per 112 000. Most (84%) of these dentists are in the two biggest cities in Cameroon: Douala and Yaoundé, which are the economic nerve centre and seat of government, respectively. The rest of the country shares just 27 (15.6%) dentists [20]. Cameroon’s disadvantaged and marginalized populations, as in other countries, bear the burden of the greatest portion of oral disease [21]. The vast majority of Cameroon’s dentists are in private practice, putting them out of financial reach of the ordinary person. Most dental clinics/offices are privately owned in the capitals of the Centre and Littoral regions, Yaoundé and Douala, respectively. Most public/government dental clinics/offices are also located in these two regions. Many Cameroonians travel several hours and sometimes days to get to the nearest dentist. It is estimated that only five of Cameroon’s 172 dentists work in rural areas, putting the ratio of dentists to population in rural areas at 1:1 900 000 and in urban areas at 1:60 000.

### Dental training

Cameroon does not have a dental school, but there are advanced plans to start at least two government-owned dental schools at the University of Yaoundé and the University of Buea. A private dental school is also planned for the University of Montaigne. Given the distribution of dentists and their limited accessibility to the poor and rural people of Cameroon, there is no assurance that opening dental schools and graduating more dentists will increase population-based oral health in Cameroon. Furthermore, Cameroon lacks the necessary human resources to establish a dental school. For example, there are currently no dentists with graduate qualifications in the dental specialties to serve as faculty. In addition, the capitalization of the planned government schools is weak, and plans to open schools have been delayed.

### Licensing of mid-levels

Outside the BCHB, there is an aging group of MLPs in 10 regional hospitals located in the capitals of each of the 10 regions. While the Cameroon National Dental Council had no licensure procedure for these practitioners, they were trained in the Yaoundé University Medical School in the 1980s specifically for the government-owned regional hospitals. The training did not continue after the first group graduated, and most are getting close to retirement age. The Dental Council does not have documentation for the number of dental assistants working with private dentists.

### Mid-level provider training

In the short term it may be more feasible to invest in a school for MLPs, rather than fully trained dentists, requiring less specialized dental manpower. MLPs for dental care typically require just 1 to 3 years of training, compared with 5 or 6 years for dentists. Many sub-Saharan African countries have utilized mid-level personnel in basic diagnosis, medical treatment, and specialties such as ophthalmology and anaesthesia. Recruitment and training of mid-level professionals resulted in low training costs, reduced training duration, and success in rural placement [22]. In Malawi, MLPs in emergency obstetrics and neonatal care are reducing the impact of a brain drain of skilled personal in this sector and improving access in rural areas [23]. In Tanzania, Mozambique, and Uganda, MLPs providing surgical and anaesthetic services in district hospitals are performing roles normally provided by specialist doctors [24]. Currently, however, Cameroon does not have any standardized licensure for MLPs. Private dentists train nurses in-house to perform dental assisting work. Organizations such as the Cameroon BCHB have established private schools to train personnel for their needs.

### Limitations

Data collection was generally consistent throughout the clinics, with a few exceptions. The registries followed a standardized clinical practice but the contents of the columns were not specifically defined in a policy. That said, these registries followed acceptable standards of recording clinical practice and are felt to accurately reflect the clinical activities and associated practitioners at these clinics. There were no data on dentures and root canals for Clinic 1. As this was one of the leading dental clinics in our sample, this was unfortunate. Data on how many procedures were done by MLPs with dentist guidance were not fully captured. We do know the dentists in these clinics teach and guide mid-level practitioners through procedures.

We did not capture any data about dental care quality. It will be important to know how durable the procedures done by MLPs are. We do not know how many patients are coming back with complications of tooth extractions, failed fillings, broken dentures, and so on, which may have resulted from poor management. The next studies of the Baptist model should focus on the comparative quality of care.

## Conclusion

The BCHB, with its minimal resources, has been able to train and employ 42 MLPs. These MLPs are delivering a significant amount of health care in Cameroon. They are relieving pain from toothaches, managing facial trauma, providing affordable removable dentures and, perhaps most importantly, encouraging prevention of oral diseases through the school oral health programme. We have shown that, with the exception of major facial injuries, MLPs are performing at the same level as the dentists and practicing the kind of dentistry that is practiced generally in Cameroon. These MLPs are definitely not providing the complex cosmetic, prosthodontics, or orthodontic procedures that dentists in the developed world are known for, but they are doing numerous extractions and fillings along with community oral health programming to prevent oral diseases. A low-income country like Cameroon, without sufficient material and human resources in oral health, should utilize a primary health-care approach to dentistry with a particular focus on MLPs providing the bulk of the workforce. The four dentists working in the BCHB are playing the vital role of training, guiding, and performing complex procedures, and their reach is maximally extended by MLPs.

The vast majority of health facilities in Cameroon, especially those run by the government, do not have dental services. Many health facilities, especially in rural areas that may never have dentists, could benefit from services provided by MLPs. The BCHB model could be formalized and replicated in Cameroon’s 166 public district hospitals [25], along with several missionary hospitals. This model is also consistent with the WHO global recommendations and guidelines on task shifting [26]. The planned dental schools that are currently stalled as a result of the lack of specialist dentist faculty could start by training MLPs. Available general dentists can effectively train mid-level providers in how to perform simple extractions and fillings. A fully functional mid-level training programme could enable Cameroon to significantly reduce the burden of dental caries.

## Abbreviations

BCHB: Baptist Convention Health Board; MLP: mid-level dental provider; WHO: World Health Organization.

## Competing interests

The authors declare that they have no competing interests.

## Authors’ contributions

LNA conceived the study and design, acquired data, and participated in analysis and integration of data, and drafting the manuscript. AMA participated in conceiving the study, and acquisition and integration of data. AH, AD, and SB participated in design and coordination, and helped to draft and edit the manuscript. All authors read and approved the final manuscript.
